# Causes, Consequences, and Preventive Strategies for Childhood Obesity: A Narrative Review

**DOI:** 10.7759/cureus.64985

**Published:** 2024-07-20

**Authors:** Ashish Goel, Spoorti Reddy, Paula Goel

**Affiliations:** 1 Department of Cardiology, Fayth Clinic, Mumbai, IND; 2 Department of General Medicine, Jan Sewa Hospital, Tantia University, Sri Ganganagar, IND; 3 Department of Pediatrics, Fayth Clinic, Mumbai, IND

**Keywords:** lifestyle intervention, developing nation, adolescent obesity, obesity, childhood obesity prevalence

## Abstract

Childhood obesity is a complex public health challenge with profound implications for both physical and psychological well-being. A significant portion of the global population struggles with obesity. Sedentary lifestyles, increased consumption of ultra-processed foods, and socioeconomic disparities are major contributors. The COVID-19 pandemic has further exacerbated these issues, leading to a surge in obesity rates among children. The consequences of childhood obesity extend beyond immediate health issues like type 2 diabetes and cardiovascular diseases; obese children are at higher risk for psychological problems such as depression, anxiety, and low self-esteem, which can persist into adulthood. These health challenges also impose substantial economic burdens due to increased healthcare costs and reduced productivity. This paper synthesizes findings from various articles to provide an overview of the causes, consequences, and preventive strategies related to childhood obesity. It highlights the varied nature of obesity, including genetic, environmental, and lifestyle factors, and discusses the profound impact on physical health, socioemotional skills, and mental health. Additionally, it examines the global challenge of childhood obesity, particularly in developing nations, and emphasizes the importance of preventive measures, family and parental behaviors, and effective policy interventions.

## Introduction and background

Childhood obesity has emerged as one of the most pressing public health issues of our time. As of February 2024, the World Health Organization estimates that 39 million children under the age of five and 340 million schoolchildren and adolescents (aged 5-19) are living with overweight or obesity, with this number continuing to rise [1]. From 1975 to 2016, the global age-standardized prevalence of obesity in children and adolescents (5-19 years) increased from 0.7% to 5.6% in girls and 0.9% to 7.8% in boys. This escalating issue is largely driven by modern sedentary lifestyles, increased screen time, the consumption of ultra-processed foods, and the prolonged impacts of the COVID-19 pandemic. In the United States, the Centers for Disease Control and Prevention report that approximately 14.7 million children and adolescents are affected by obesity, with significant disparities observed across different racial and socioeconomic groups. The issue is not confined to high-income countries but is also prevalent in low- and middle-income nations, particularly in urban settings. Seventy percent of children with obesity and overweight live in low- and middle-income countries. In Africa, the number of overweight children under five has surged by nearly 23% since 2000. Additionally, the increase in childhood obesity is not uniform across all demographics; boys tend to have higher rates of overweight and obesity compared to girls, and this trend is expected to continue, with projections showing a significant rise by 2030. Low- and middle-income countries are witnessing a faster growth rate of childhood obesity compared to high-income countries, suggesting a shifting burden of disease. Factors such as genetic predisposition, environmental influences, dietary habits, physical inactivity, and socioeconomic status all contribute to the development of obesity in children [2,3]. The impact of childhood obesity extends beyond immediate physical health problems; obese children are at a higher risk of developing severe long-term health issues, including type 2 diabetes, cardiovascular diseases, and certain types of cancer [4,5]. Additionally, the psychological effects of obesity, such as low self-esteem, depression, and anxiety, can significantly impair a child's quality of life and socioemotional development [6,7].

This paper aims to provide an overview of the causes and consequences of childhood obesity. It discusses various preventive strategies and interventions that may help mitigate the impact of obesity on children and society as a whole.

## Review

Causes of childhood obesity

The development of childhood obesity is influenced by a multitude of factors, including genetic predisposition, environmental influences, dietary habits, physical inactivity, and socioeconomic status. Early predictors of obesity include maternal obesity during pregnancy, excessive gestational weight gain, and gestational diabetes, all of which are associated with increased birth weight. A systematic review by Jebeile et al. indicates that breastfeeding has a protective effect against the development of childhood obesity. Conversely, introducing complementary foods and beverages before the age of four months, particularly in formula-fed infants, is associated with a higher likelihood of obesity [8].

Children with obese parents are more likely to become obese themselves due to inherited genetic factors. Most instances of obesity are polygenic, meaning multiple genes contribute small effects to the overall phenotype. In contrast, genetic variants that significantly impact body weight are rare but notable for their extreme phenotypes, often manifesting as early-onset and severe obesity, as demonstrated by Haqq et al. [9]. However, genetic factors alone do not account for the rapid increase in obesity rates. The interaction between genetic predisposition and environmental factors is crucial in understanding the development of obesity [2,3].

Environmental factors play a significant role in the prevalence of childhood obesity. The availability of unhealthy food options and a lack of safe spaces for physical activity contribute to rising obesity rates. Urbanization has led to a reduction in physical activity spaces and an increase in sedentary lifestyles [10,11]. The consumption of high-calorie, low-nutrient foods combined with sedentary lifestyles, such as excessive screen time, promotes weight gain. Fast-food restaurants and the easy availability of processed foods further exacerbate unhealthy eating habits among children [12,13]. Additionally, increased screen time and the popularity of video games have significantly reduced the time children spend in physical activities [14].

Socioeconomic status is another critical factor influencing childhood obesity. Children from lower socioeconomic backgrounds are more likely to be obese due to limited access to healthy foods and recreational activities. Socioeconomic factors also influence parental knowledge and attitudes toward nutrition and physical activity [15,16]. Parental behaviors and the home environment significantly impact children's eating habits and activity levels. Parents who consume unhealthy diets and lead sedentary lifestyles are more likely to have obese children. Moreover, parental stress and its management play a crucial role in shaping children's eating behaviors and physical activity levels [17-19].

Cultural attitudes toward body weight and food play a role in the development of childhood obesity. In some cultures, a higher body weight is associated with wealth and prosperity, which can hinder efforts to promote a healthy body weight. Psychological factors, such as emotional eating and coping mechanisms, further contribute to the development of obesity [20,21].

Physical health consequences

In the short term, obesity in children leads to conditions such as type 2 diabetes, hypertension, and dyslipidemia, which were once considered adult illnesses [1,4]. These conditions not only affect the child's current health but also set the stage for chronic health problems in adulthood. The chronic nature of obesity-related health issues, including cardiovascular diseases and metabolic syndrome, emphasizes the need for early intervention. Obese children are more likely to become obese adults, perpetuating a cycle of health problems [5-7]. Childhood obesity increases the risk of developing noncommunicable diseases (NCDs) later in life, including diabetes, cardiovascular diseases, and certain cancers [16-17]. Studies have shown that childhood obesity is associated with an increased risk of early mortality from various causes.

A study by Franks et al. followed nearly 5,000 American Indian children over more than two decades and found that childhood obesity, glucose intolerance, and hypertension significantly increase the risk of premature death from endogenous causes [22]. The long-term mortality risk further highlights the critical need for early intervention and sustained preventive measures. The systematic review by Reilly and Kelly found that childhood and adolescent overweight and obesity are significantly linked to increased risks of premature mortality and various adult morbidities, including cardiometabolic diseases such as diabetes, hypertension, ischemic heart disease, and stroke, as well as conditions like asthma, disability pension, and symptoms of polycystic ovary syndrome [23].

Moreover, multiple organ systems may be affected by childhood obesity, leading to conditions such as fatty liver disease, orthopedic problems, and respiratory issues. Metabolic dysfunction-associated steatotic liver disease, or metabolic dysfunction-associated steatohepatitis, is increasingly diagnosed in obese children and can progress to liver cirrhosis. Orthopedic problems, such as Blount's disease and slipped capital femoral epiphysis, are also more common in obese children due to the increased stress on weight-bearing joints [13,24,25].

Psychological and socioemotional impact

Obese children have an increased risk of depression, anxiety, and low self-esteem, with these psychological issues often persisting into adulthood and affecting overall quality of life [12,26]. Puhl and Latner [26] found that the stigma and discrimination associated with obesity further exacerbate these psychological challenges, leading to social isolation and mental health concerns. Weight stigma often results in internalized weight-biased beliefs and attitudes, where individuals accept and apply negative stereotypes to themselves. Latner and Stunkard reported that the stigmatization of obese children has worsened over time, contributing to these psychological issues [27]. Eisenberg et al. found that weight-based teasing is strongly associated with poorer emotional well-being among adolescents [28].

Additionally, childhood obesity often impacts socioemotional development, leading to difficulties in social interactions and academic performance [29,30]. Obese children experience bullying and social exclusion, negatively affecting their self-esteem and academic achievement [6,7]. Lumeng et al. [31] found that weight status is a predictor of being bullied in school, which can have long-lasting effects on mental health and socioemotional development. Similarly, Puhl and Heuer [32] emphasized that the emotional distress from bullying often leads to a vicious cycle where stress eating contributes to further weight gain.

Sleep disturbances are also common among obese children. Obesity is linked to sleep disorders such as obstructive sleep apnea, which can affect overall health and cognitive functioning. Owens [33] reported that poor sleep quality is prevalent among obese children, contributing to weight gain and perpetuating another vicious cycle. Similarly, Must and Strauss [34] highlighted the significant health risks and consequences of childhood and adolescent obesity, including sleep disorders.

Pediatric weight stigma negatively affects parents and guardians as well. Families often report feelings of isolation, blame, and fear for their child's health and weight challenges. Parents with their own weight issues tend to internalize self-blame for their child's weight, leading to frustration and inadequacy. It is crucial to consider the holistic consequences of weight stigma, encompassing both pediatric and parental experiences [9].

Socioeconomic impact

Increased healthcare costs are associated with treating obesity-related conditions in children, placing a strain on healthcare systems worldwide. Treating obesity and its related health conditions requires substantial medical resources, including frequent doctor visits, medications, and sometimes surgical interventions. Finkelstein et al. [35] estimated that the lifetime direct medical costs of childhood obesity can be significantly compared to children of healthy weight. These costs are associated with treating obesity-related conditions such as type 2 diabetes, cardiovascular diseases, and other comorbidities. Similarly, Cawley and Meyerhoefer [36] revealed that obesity significantly increases medical expenses, with obese individuals incurring higher healthcare costs compared to non-obese individuals. The study highlighted that the medical costs of obesity are not only a result of direct treatment for obesity-related conditions but also from increased utilization of healthcare services.

Childhood obesity can lead to decreased productivity and increased absenteeism in schools and later in the workplace, further exacerbating the economic burden on society. Obese children are more likely to miss school due to health issues, which can affect their educational attainment and future employment opportunities. Van Jaarsveld and Gulliford [37] observed trends in childhood obesity from primary care electronic health records in England, noting that obesity can significantly affect a child's school attendance and performance. Similarly, Halfon and Hochstein [38] emphasized how early health experiences affect long-term health outcomes. They highlighted that childhood obesity can have lasting effects on an individual's health trajectory, influencing both physical and mental health into adulthood. Childhood obesity can, therefore, lead to educational challenges and reduced occupational opportunities, which persist into adulthood.

Beyond healthcare costs, indirect costs include lost productivity, reduced earning potential, and increased disability claims. Addressing childhood obesity can, therefore, have significant economic benefits for society. The systematic reviews by Knai et al., Waters et al., and Brown and Summerbell highlighted that interventions, especially school-based programs involving dietary changes and physical activity promotion, are effective in preventing childhood obesity [39-41]. These programs, which also involve parental and community engagement, can lead to long-term health benefits and significant economic savings by reducing future healthcare costs associated with obesity.

Rising prevalence in developing nations: a case study of India

The prevalence of childhood obesity is rising not only in developed nations but also in developing countries, posing new challenges and amplifying health and socioeconomic impacts in these regions. Countries like India are experiencing a surge in childhood obesity rates due to urbanization, changing dietary patterns, and reduced physical activity. The rising obesity rates in developing nations can be linked to the adoption of Western diets and lifestyles, which increase the risk of NCDs [27]. Traditionally, Indian diets were rich in complex carbohydrates and fiber; however, these are quickly being replaced by diets high in fats, sugars, and processed foods. Coupled with increased screen time and sedentary lifestyles, these factors contribute to rising obesity rates. A two-decade meta-analysis by Singh et al. found the prevalence rate of childhood obesity to be 8.4%, while the prevalence of childhood overweight was 12.4% [42]. Male children tend to have a higher risk of developing obesity compared to female children. Urban and high-income regions of the country, including Mumbai and Delhi, were found to have a significantly higher prevalence of childhood obesity, although recent times also indicate a disproportionate increase in rural and urban low-income populations. Further studies are required to comprehensively study these trends, but current data indicate a rising rate of obesity as the gross domestic product increases, a phenomenon also seen in other parts of the world. The effects of this are multifold. The immediate impact observed was an increase in hypertension rates among school children, including those from lower socioeconomic backgrounds, as demonstrated in a study by Goel and Goel [43]. Long-term effects such as insulin resistance, cardiovascular disease, and an increased incidence of bariatric surgery have been observed. This trend is alarming and highlights the need for urgent intervention [18]. The healthcare infrastructure in developing nations may be ill-equipped to handle this growing burden, leading to increased morbidity and mortality rates [44,45]. Furthermore, childhood obesity can exacerbate poverty cycles by reducing educational attainment and future earnings [15]. This epidemic has prompted the Health Ministry of the Government of India to mandate and incentivize multiple initiatives to combat childhood obesity. Experienced professionals have drafted (in collaboration with both the public and private sectors) and implemented programs across the country with the specific goal of increasing general awareness and creating healthier outcomes for its citizens.

Preventive strategies and interventions

Parents play a crucial role in shaping children's dietary habits and physical activity levels. Interventions targeting parental education and involvement are, therefore, essential. A randomized trial by Haines et al. found that encouraging consistent household routines, specifically by extending sleep duration and limiting TV watching, may be a successful strategy for lowering body mass index in low-income, racial/ethnic minority children [46]. Similarly, Kitzman-Ulrich et al. [47] emphasized that parenting programs focusing on healthy eating habits, improved sleep, and active lifestyles can be very effective in preventing childhood obesity.

Furthermore, implementing comprehensive school-based programs that promote healthy eating and physical activity significantly reduces obesity rates. These programs include nutrition education, physical education, and healthier school meal options. Schools serve as critical settings for early intervention, providing children with the knowledge and skills to make healthy choices. Story et al. [48] found that creating healthy food and eating environments through policy and environmental approaches is essential for promoting health in schools. Gortmaker et al. [49] highlighted that school-based programs provide children with the knowledge and skills to make healthy choices, leading to reduced obesity rates.

Public policies aimed at regulating food marketing to children, improving food labeling, and creating healthier food environments are beneficial. Effective policies can help create a supportive environment for children to develop healthy habits. Sassi et al. [50] and Cecchini et al. [51] found that policies such as taxation on sugary drinks and junk food, along with subsidies for healthier options, drive positive changes in dietary habits. Community programs that promote physical activity and provide access to healthy foods can support families in making healthier lifestyle choices. Community gardens, local farmers' markets, and safe recreational spaces encourage healthier living [20,21]. Wang et al. [52] demonstrated that improved food environments, including community gardens, local farmers' markets, and safe recreational spaces, help prevent childhood obesity.

Healthcare providers play a key role in the early detection and management of childhood obesity. Regular screenings, counseling, and follow-up can help identify at-risk children and provide timely interventions [38,53]. Dietz and Robinson highlighted the importance of healthcare providers in managing obesity through regular screenings and counseling [53]. Leveraging technology, such as mobile health applications and telemedicine, supports ongoing monitoring and personalized interventions for obese children. Epstein et al. found that these tools help track dietary habits and physical activity, providing tailored feedback for better management [54].

Behavioral support strategies in obesity management should address dietary intake, physical activity, sedentary behaviors, sleep hygiene, and behavioral components within a family-centered, age-appropriate approach aimed at fostering lasting behavior changes. Customizing interventions for various subgroups based on age, gender, and culture may be necessary. For instance, therapy for young children might be largely parent-focused, whereas adolescents might require a greater degree of autonomy [8].

The Etiology-Based Personalized Intervention Strategy Targeting Childhood Obesity (EPISTCO) model introduces a personalized intervention strategy that integrates nutritional, lifestyle, and physical activity approaches. These interventions are customized, prioritized, prescribed, and supervised according to individual needs across various environments. Motevalli et al. [55] emphasized that this approach aims to address the shortcomings of previous childhood obesity interventions by offering a comprehensive strategy, with implementation varying based on specific circumstances.

Addressing childhood obesity requires comprehensive strategies that encompass individual, family, community, and policy-level interventions. Collaborative efforts among various stakeholders are necessary to create supportive environments that promote healthy behaviors and prevent obesity.

Case studies and successful interventions

The rising rates of childhood obesity have prompted public health organizations around the world to take action and mandate initiatives.

In 2012, the Indian Academy of Pediatrics (IAP) initiated the TASK Force on Obesity in Children and Adolescents, which continued until 2017. In 2017, the Noncommunicable Disease Pediatric Academy was established as a subspecialty chapter of IAP. In 2022, under the auspices of that year's IAP president, the chapter launched the “Sankalp: Swasthya Sampoorna” initiative [56]. This initiative aimed to raise awareness of NCDs in schools and colleges. Modules were developed on various topics, including a healthy lifestyle, balanced nutrition, reading food labels, sleep, physical activity, gadget addiction, alcohol and smoking prevention, mental health, and environmental pollution. The chapter conducts monthly webinars, seminars, and projects on different aspects of NCD prevention. Additionally, a healthy recipe contest is organized to encourage healthy eating within the community.

Singapore has implemented a comprehensive strategy to tackle childhood obesity that includes school-based interventions, community programs, and policy measures. The "Healthy Meals in Schools Programme" ensures that school canteens provide healthier meals and regular physical activity is incorporated into the school curriculum. Additionally, public health campaigns promote healthy eating and active living among children and their families [57].

Mexico has introduced several policy measures to address childhood obesity, including taxes on sugary drinks and junk food, restrictions on marketing unhealthy foods to children, and mandatory labeling of processed foods. These measures have shown promising results in reducing the consumption of unhealthy foods and beverages among children [58].

The United States has implemented various school-based programs, such as the "Let's Move!" initiative, which aims to promote physical activity and healthy eating among children. Additionally, community-based programs and policy measures, such as improved food labeling and restrictions on junk food marketing, have been introduced to support healthy behaviors [59,60].

Finland has successfully reduced childhood obesity rates through a comprehensive approach that includes school-based interventions, community programs, and national policies. The "Schools on the Move" program encourages physical activity during school hours, while public health campaigns promote healthy eating habits. Additionally, national policies support the availability of healthy foods and create environments that encourage physical activity [61].

Pharmacotherapy and surgical intervention

Pharmacotherapy for obesity has evolved significantly over the years, reflecting advancements in understanding obesity's complex etiology and the body's response to various treatments. Historically, pharmacological treatments for obesity have included a range of drugs aimed at reducing appetite, increasing metabolism, or inhibiting fat absorption. Initial treatments were often associated with significant side effects and limited efficacy, but ongoing research has led to the development of more effective and safer medications.

Today, several FDA-approved medications are available for treating obesity in children and adolescents. These medications are designed to complement lifestyle modifications such as diet and exercise and are typically reserved for cases where such modifications alone have not yielded significant results. Orlistat is one such medication that inhibits intestinal lipase, reducing the absorption of dietary fats by approximately 30%. Approved in 2003 for use in children aged 12 and older, Orlistat's common side effects include gastrointestinal issues such as flatulence, fecal urgency, and steatorrhea, which can limit its long-term use [62]. Another medication, phentermine, is a central norepinephrine uptake inhibitor and a nonselective serotonin and dopamine reuptake inhibitor, which suppresses appetite by activating POMC neurons in the lateral hypothalamus. Approved for short-term use in individuals over 16 years old, its applicability is limited for most adolescents [62,63]. Topiramate, though not FDA-approved for weight loss, is used off-label due to its potential to suppress appetite and induce weight loss. Combined with extended-release phentermine, it was FDA-approved in 2022 for significant weight loss in adolescents aged 12-17 who have not succeeded with lifestyle modifications alone [63]. Liraglutide is another important medication, a glucagon-like peptide-1 (GLP-1) receptor agonist that reduces appetite and food cravings, increases satiety, and alters food reward pathways. Approved in 2020 for use in adolescents, it is not recommended for patients with a personal or family history of certain thyroid cancers [63]. Setmelanotide is a melanocortin-4 receptor agonist approved for obesity due to specific genetic disorders, including POMC, PCSK1, and LEPR deficiency, and Bardet-Biedl syndrome [63,64].

For cases of severe obesity, metabolic and bariatric surgery remains an option. Procedures like vertical-sleeve gastrectomy and laparoscopic Roux-en-Y gastric bypass have been shown to achieve significant and sustained weight loss. These procedures can result in a BMI reduction of 10-17 kg/m² after three to five years, with improvements in conditions like type 2 diabetes and dyslipidemia [65]. However, potential complications include the need for re-operations, gallbladder stones, and micronutrient deficiencies, highlighting the importance of both short- and long-term monitoring [65,66]. While pharmacotherapy and bariatric surgery present effective interventions for treating obesity in children and adolescents, they should be viewed as part of a broader, multi-faceted approach that includes lifestyle modifications, behavioral therapy, and supportive environments. Additionally, cost and availability are important factors that dictate their use in developing nations [67].

Future directions and research

Addressing childhood obesity requires ongoing research to understand its causes and develop effective interventions. Future research should focus on several key areas. Longitudinal studies that track children over time are crucial, as they provide valuable insights into the development of obesity and the effectiveness of interventions. These studies can help identify critical periods for intervention and the long-term impact of early-life behaviors on obesity risk. For example, understanding the influence of infant feeding practices, physical activity patterns, and dietary habits over time can highlight when and how to intervene effectively [23,68,69]. Guidelines can then be developed on nutrition, physical activity, behavioral changes, and the role of family involvement in managing and preventing obesity. Research into the interactions between genetic predisposition and environmental factors is also essential to help identify children at higher risk of obesity and inform personalized interventions. Genetic studies can reveal how specific genes influence susceptibility to obesity, while environmental research can examine factors such as diet, physical activity, and socioeconomic status. Understanding how these factors interact can shed light on the mechanisms underlying obesity development and the creation of targeted strategies [70].

Evaluating the effectiveness of various interventions is vital to identifying best practices for preventing and managing childhood obesity. This includes assessing the impact of school-based programs, community initiatives, policy measures, and technological interventions. For instance, school-based programs that promote healthy eating and physical activity can be compared to community initiatives that provide access to healthy foods and safe places for exercise. Policy measures, such as sugary drink taxes and food labeling regulations, can also be evaluated for their effectiveness in reducing obesity rates [71]. Finally, research should focus on addressing health disparities related to childhood obesity. This includes understanding how socioeconomic status, race, and ethnicity influence obesity risk and developing targeted interventions to support vulnerable populations. For example, children from low-income families may have limited access to healthy foods and safe places for physical activity, increasing their risk of obesity. While it may seem counterintuitive, undernutrition can coexist with obesity, particularly in populations with limited access to nutrient-dense foods. This phenomenon is referred to as the "double burden of malnutrition" [72].

Low-income families may face barriers to accessing healthy foods, leading to diets high in refined carbohydrates, sugars, and unhealthy fats while lacking essential vitamins and minerals. This may result in children being both overweight and undernourished, as they consume enough calories but not the necessary nutrients for healthy growth and development. Additionally, low-income families may live in areas with poor access to sunlight, which precipitates vitamin D deficiency. Vitamin D deficiency has been linked to an increased risk of obesity in children. It may influence the development of obesity through various mechanisms, including its role in calcium metabolism, insulin sensitivity, and inflammatory processes. Zakharova et al. [73] found that vitamin D insufficiency was more prevalent in children with a higher BMI, suggesting a strong correlation between obesity and lower vitamin D. By studying these disparities, researchers can then develop strategies to ensure all children have the opportunity to maintain a healthy weight, regardless of their background [74,75].

## Conclusions

Childhood obesity is a complex issue with far-reaching effects on physical health, psychological well-being, and socioeconomic factors. A comprehensive understanding of its nature is essential for developing effective prevention and intervention strategies. Collaborative efforts involving various stakeholders are being implemented to address this growing epidemic and improve the health and well-being of future generations. The increasing prevalence of childhood obesity, particularly in developing nations, emphasizes the need for global action and effective policy implementation to curb this public health crisis. A concerted effort is required from families, communities, schools, healthcare providers, and policymakers. While intervention projects have been successfully implemented globally, a greater effort is required to roll out these programs outside of urban and tier I cities, where the bulk of childhood obesity actually lies. Policymakers must prioritize creating environments that support healthy lifestyles and reduce the barriers to accessing nutritious foods and physical activity opportunities. Broader social determinants of health, including education, income inequality, and access to healthcare, need to be thoroughly addressed. Since childhood obesity strongly predicts adult weight status and health outcomes, early intervention, education, and supportive environments are paramount. By implementing evidence-based strategies and fostering a culture of health, we can try to mitigate the impact of childhood obesity and ensure a healthier future for future generations.
